# The Accuracy and Appropriateness of ChatGPT Responses on Nonmelanoma Skin Cancer Information Using Zero-Shot Chain of Thought Prompting

**DOI:** 10.2196/49889

**Published:** 2023-12-14

**Authors:** Ross O'Hagan, Dina Poplausky, Jade N Young, Nicholas Gulati, Melissa Levoska, Benjamin Ungar, Jonathan Ungar

**Affiliations:** 1 Department of Dermatology Icahn School of Medicine at Mount Sinai New York, NY United States

**Keywords:** ChatGPT, artificial intelligence, large language models, nonmelanoma skin, skin cancer, cell carcinoma, chatbot, dermatology, dermatologist, epidermis, dermis, oncology, cancer

## Introduction

Nonmelanoma skin cancer (NMSC) represents the most prevalent form of cancer worldwide [1]. Patients with NMSC seek information from various resources. Work has already shown that language learning models (LLMs) such as ChatGPT can generate medical information in response to questions [2]; however, results vary significantly based on the prompts entered. Previous work has shown that a few-shot approach, where one provides several example prompts and outputs, has good results [3], as does the few-shot chain of thought approach, where answers include examples and the reasoning for correct answers, encouraging the model to reason through the question [4]. Zero-shot chain of thought (ZS-COT) prompting does not provide example prompts; instead, it uses phrases to encourage the LLMs to “think” through their responses, with significant improvement in accuracy in some contexts [5]. In this study, we explore ChatGPT’s performance in answering questions about NMSC using both standard and ZS-COT prompting.

## Methods

### Overview

**Figure 1:**
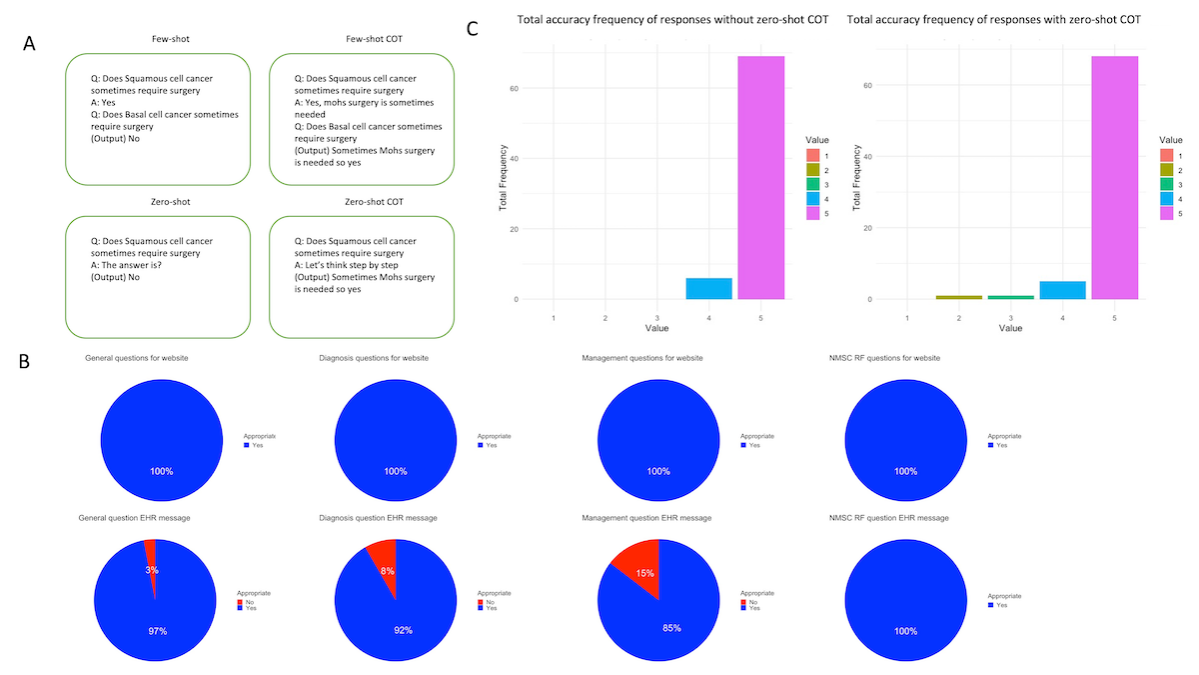
(A) Example of several popular language learning model prompting techniques. (B) Percent of appropriate responses for each question category by medium. (C) Accuracy scores by prompt style. COT: chain of thought; EHR: electronic health record; NMSC: nonmelanoma skin cancer; RF: risk factor.

We generated 25 common clinical questions about NMSC in four categories: general, diagnosis, management, and risk factors. Prompts were entered into ChatGPT 4.0 on March 31, 2023, and responses were recorded for both standard and ZS-COT prompting (Figure 1A). Ending ZS-COT prompting queries with “Let’s think step by step” has been shown to improve performance in previous papers [5]. Three attending dermatologists independently reviewed and graded whether the outputs would be appropriate for a patient-facing website and an electronic health record (EHR) message draft to a patient. Responses were also evaluated for accuracy on a 5-point scale, with 1 being completely inaccurate and 5 being completely accurate, and reviewers assessed which of the two prompting styles they preferred. Statistical differences between prompts were computed using the Wilcoxon test. Statistical analysis was performed in R version 4.2.2 (R Foundation for Statistical Computing).

### Ethical Considerations

This study did not require institutional review board approval.

## Results

Averaging all accuracy scores from a scale (range 1-5), we found that the combined accuracy for both the original prompt and ZS-COT prompt was 4.89. The average accuracy score from all 25 questions asked for the original prompt and ZS-COT prompt was 4.92 and 4.87, respectively, representing a nonsignificant difference of 1.03%. Both models were deemed 100% appropriate for a patient-facing information portal for general, diagnosis, management, and risk factor questions. For EHR message responses, outputs were appropriate for 97% of general questions, 92% of diagnosis questions, 85% of management questions, and 100% of risk factor questions (Figure 1B). The lowest accuracy grade for the standard prompting responses and ZS-COT prompting was 4 and 2, respectively (Figure 1C). This score was given for the prompt “What causes basal cell carcinoma?” (Multimedia Appendix 1).

## Discussion

This exploratory qualitative study found that LLMs can provide accurate patient information regarding NMSC appropriate for both general websites and EHR messages. We found that ZS-COT prompting does not provide more accurate dermatology information. The limitations of this study include that we only explored a subset of clinical questions patients may have about NMSC, there is no objective standard for appropriateness, and the personal biases of the dermatologists may bias response preference. As LLMs continue to grow and be adapted, clinicians must monitor their clinical utility and how different prompting methods may change the quality of results.

## Appendix

Multimedia Appendix 1 Evaluated nonmelanoma skin cancer questions.

## Abbreviations

EHR: electronic health record
LLM: language learning model
NMSC: nonmelanoma skin cancer
ZS-COT: zero-shot chain of thought
