# Supplementary material for: The Accuracy and Appropriateness of ChatGPT Responses on Nonmelanoma Skin Cancer Information Using Zero-Shot Chain of Thought Prompting
Source: JMIR Dermatol. 2023 Dec 14;6:e49889. doi: 10.2196/49889 (PMC10755659; doi:10.2196/49889)
Supplement: Multimedia Appendix 1 [file derma_v6i1e49889_app1.docx]

Supplemental Table 1: Evaluated non-melanoma skin cancer questions

| **Question Category** | **Questions without Zero-shot COT** | **Questions with**  **Zero-shot COT** |
| --- | --- | --- |
| General Questions | What is basal cell carcinoma? | Q: What is basal cell carcinoma?  A: Let's think step by step |
| General Questions | What is squamous cell carcinoma? | Q: What is squamous cell carcinoma?  A: Let's think step by step |
| General Questions | What causes basal cell carcinoma? | Q: What causes basal cell carcinoma?  A: Let's think step by step |
| General Questions | What causes squamous cell carcinoma? | Q: What causes squamous cell carcinoma? A: Let's think step by step |
| General Questions | How are basal cell carcinoma and squamous cell carcinoma different? | Q: How are basal cell carcinoma and squamous cell carcinoma different?  A: Let's think step by step |
| General Questions | Can basal cell carcinoma kill you? | Q: Can basal cell carcinoma kill you?  A: Let's think this step by step |
| General Questions | Can squamous cell carcinoma kill you? | Q: Can squamous cell carcinoma kill you?  A: Let's think step by step |
| General Questions | Can basal cell carcinoma spread to other parts of the body? | Q: Can basal cell carcinoma spread to other parts of the body?  A: Let's think step by step |
| General Questions | Can squamous cell carcinoma spread to other parts of the body? | Q: Can squamous cell carcinoma spread to other parts of the body?  A: Let's think step by step |
| General Questions | Is basal cell carcinoma genetic? | Q: Is basal cell carcinoma genetic?  A: Let's think step by step |
| General Questions | Is squamous cell carcinoma genetic? | Q: Is squamous cell carcinoma genetic?  A: Let's think step by step |
| Diagnosis Questions | What does basal cell carcinoma look like? | Q: What does basal cell carcinoma look like?  A: Let's think step by step |
| Diagnosis Questions | What does squamous cell carcinoma look like? | Q: What does squamous cell carcinoma look like?  A: Let's think step by step |
| Management Questions | How is basal cell carcinoma treated? | Q: How is basal cell carcinoma treated?  A: Let's think step by step |
| Management Questions | How is squamous cell carcinoma treated? | Q: How is squamous cell carcinoma treated?  A: Let's think step by step |
| Management Questions | Can basal cell carcinoma be treated without surgery? | Q: Can basal cell carcinoma be treated without surgery?  A: Let's think step by step |
| Management Questions | Can squamous cell carcinoma be treated without surgery? | Q: can squamous cell carcinoma be treated without surgery?  A: Let's think step by step |
| Management Questions | What is Mohs surgery? | Q: What is Mohs surgery?  A: Let's think step by step |
| Management Questions | What are the risks of skin cancer surgery? | Q: What are the risks of skin cancer surgery?  A: Let's think step by step |
| Management Questions | Do I need chemotherapy for basal cell carcinoma? | Q: Do I need chemotherapy for basal cell carcinoma?  A: Let's think step by step |
| Management Questions | Do I need chemotherapy for squamous cell carcinoma? | Q: Do I need chemotherapy for squamous cell carcinoma?  A: let's think step by step |
| NMSC Risk Factor Questions | Will sunscreen protect me from getting basal cell carcinoma? | Q: Will sunscreen protect me from getting basal cell carcinoma?  A: Let's think step by step |
| NMSC Risk Factor Questions | Will sunscreen protect me from getting squamous cell carcinoma? | Q: Will sunscreen protect me from getting squamous cell carcinoma?  A: let's think step by step |
| NMSC Risk Factor Questions | Can I still get basal cell carcinoma if I have darker skin? | Q: Can I still get basal cell carcinoma if I have darker skin?  A: Let's think step by step |
| NMSC Risk Factor Questions | Can I still get squamous cell carcinoma if I have darker skin? | Q: Can I still get squamous cell carcinoma if I have darker skin?  A: Let's think this step by step |

NMSC stands for non-melanoma skin cancers.
